# Midwives (Scotland) Act, 1915

**Published:** 1921-02-05

**Authors:** 


### Midwives (Scotland) Act, 1915.

The following is the constitution of the Central Midwives Board for Scotland (Scottish Board of Health) as from February 1, 1921:—

Appointed by Scottish Board of Health.—Miss Alice Helen Turnbull, Superintendent Health Visitor, Maternity and Child Welfare Department, Edinburgh. Miss Isabella Lewis Scrimgeour, Superintendent, Cottage Nurses' Training Home, Govan, and Miss Kate Leslie Scott, Chairman of Council of Scottish Midwives' Association, 15 Carden Place, Aberdeen (both midwives practising in Scotland).

Appointed by Association of County Councils for Scotland.—Sir Archibald Buchan-Hepburn, Bart. (Convener of Haddington County Council), Letham, Haddington.

Appointed by Convention of Royal Burghs of Scotland.—Sir Robert Cranston, K.C.V.O., etc., 19 Merchiston Avenue, Edinburgh.

Appointed by Queen Victoria's Jubilee Institute for Nurses (Scottish Branch).—James Haig Ferguson, Esq., M.D., F.R.C.S.E., 7 Coates Crescent, Edinburgh.

Appointed by Society of Medical Officers of Health of Scotland.—Dr. A. Campbell Munro.

Appointed by University Courts of the Universities of Edinburgh and St. Andrews.—Professor J. A. C. Kynoch, Professor of Midwifery in the University of St. Andrews.

Appointed by University Courts of the Universities of Glasgow and Aberdeen. — Professor Robert Gordon McKerron, M.A., M.D., Professor of Midwifery in the University of Aberdeen, 2 Queen's Terrace, Aberdeen.

Appointed by Royal College of Physicians of Edinburgh, Royal College of Surgeons of Edinburgh, Royal Faculty of Physicians and Surgeons of Glasgow.—Dr. Robert Jardine, F.R.F.P.S. (Glasgow), 20 Royal Crescent, Glasgow.

Appointed by Scottish Committee of British Medical Association.—Dr. Michael Dewar, 5 Chalmers Street, Edinburgh; Dr. E. H. L. Oliphant, 23 Newton Place, Glasgow.

